# Comparative proteomic analysis of drought tolerance in the two contrasting Tibetan wild genotypes and cultivated genotype

**DOI:** 10.1186/s12864-015-1657-3

**Published:** 2015-06-05

**Authors:** Nanbo Wang, Jing Zhao, Xiaoyan He, Hongyan Sun, Guoping Zhang, Feibo Wu

**Affiliations:** Institute of Crop Science, Department of Agronomy, College of Agriculture and Biotechnology, Zijingang Campus, Zhejiang University, Hangzhou, 310058 People’s Republic of China; Jiangsu Co-Innovation Center for Modern Production Technology of Grain Crops, Yangzhou University, Yangzhou 225009, China, Yangzhou University, Yangzhou, 225009 China

**Keywords:** Drought stress, Barley (*Hordeum vulgare* L*)*, Proteomic, Tibetan wild barley, Mass spectrometry

## Abstract

**Background:**

Drought is one of major abiotic stresses constraining crop productivity worldwide. To adapt to drought stress, plants have evolved sophisticated defence mechanisms. Wild barley germplasm is a treasure trove of useful genes and offers rich sources of genetic variation for crop improvement. In this study, a proteome analysis was performed to identify the genetic resources and to understand the mechanisms of drought tolerance in plants that could result in high levels of tolerance to drought stress.

**Results:**

A greenhouse pot experiment was performed to compare proteomic characteristics of two contrasting Tibetan wild barley genotypes (drought-tolerant XZ5 and drought-sensitive XZ54) and *cv*. ZAU3, in response to drought stress at soil moisture content 10 % (SMC10) and 4 % (SMC4) and subsequently 2 days (R1) and 5 days (R2) of recovery. More than 1700 protein spots were identified that are involved in each gel, wherein 132, 92, 86, 242 spots in XZ5 and 261, 137, 156, 187 in XZ54 from SMC10, SMC4, R1 and R2 samples were differentially expressed by drought over the control, respectively. Thirty-eight drought-tolerance-associated proteins were identified using mass spectrometry and data bank analysis. These proteins were categorized mainly into photosynthesis, stress response, metabolic process, energy and amino-acid biosynthesis. Among them, 6 protein spots were exclusively expressed or up-regulated under drought stress in XZ5 but not in XZ54, including melanoma-associated antigen p97, type I chlorophyll a/b-binding protein b, glutathione S-transferase 1, ribulosebisphosphate carboxylase large chain. Moreover, type I chlorophyll a/b-binding protein b was specifically expressed in XZ5 (Spots A4, B1 and C3) but not in both of XZ54 and ZAU3. These proteins may play crucial roles in drought-tolerance in XZ5. Coding Sequences (CDS) of *rbcL* and *Trx-M* genes from XZ5, XZ54 and ZAU3 were cloned and sequenced. CDS length of *rbcL* and *Trx-M* was 1401 bp (the partial-length CDS region) and 528 bp (full-length CDS region), respectively, encoding 467 and 176 amino acids. Comparison of gene sequences among XZ5, XZ54 and ZAU3 revealed 5 and 2 SNPs for *rbcL* and *Trx-M*, respectively, with two 2 SNPs of missense mutation in the both genes.

**Conclusions:**

Our findings highlight the significance of specific-proteins associated with drought tolerance, and verified the potential value of Tibetan wild barley in improving drought tolerance of barley as well as other cereal crops.

**Electronic supplementary material:**

The online version of this article (doi:10.1186/s12864-015-1657-3) contains supplementary material, which is available to authorized users.

## Background

Currently, approximately 1/3 of the world’s arable land faces yield reduction due to cyclical or unpredictable drought, a great threat to agricultural production [1]. To meet the needs of the growing world population, it is essential to effectively utilize dehydrated soil in drought-prone areas. Development and planting of drought tolerant cultivars is a cost-effective and practically acceptable approach for full utilization of water-limiting soil [2]. However, the progress toward developing drought-tolerant crops is significantly hampered by the lack of high tolerant genetic resources and the complexity in physiological and genetic traits. It is therefore important to identify the genetic resources and to understand the mechanisms of drought tolerance in plants that could result in high levels of tolerance to drought stress.

Remarkable studies have been done concerning drought tolerance in cultivated crops [3]. Abiotic stress such as drought and salt stress induces changes of protein expression in plants [4]. Protein expression changes in response to drought have been reported in rice [4], maize [5], sugar beet [6], wheat [7] and sunflower [8]. A number of drought-induced proteins were identified involving in photosynthesis, signaling pathways, oxidative stress detoxification [4]. It has been reported that several proteins, with a function in the protection and repair of proteins such as the heat shock proteins (Hsp), are expressed under drought stress [9]. Pathogenesis-related (PR) proteins have also been reported to be induced by drought stress [10]. Huerta-Ocampo et al. [11] found that amaranth root response to drought stress involved the up-regulation of proteins that control damage from reactive oxygen species, a family of heat shock proteins. Comparative proteomics approaches to analyze protein abundance between normal and stress-treated or tolerant and sensitive genotypes have greatly facilitated the study of plant cellular stress responses. However, only limited information is available on drought induced specific proteins in barley.

Barley (*Hordeum vulgare* L) is one of the most widely cultivated cereals crops in the world [12]. Compared to the other cereals, barley plants, exhibiting high drought tolerance, are the most suitable targets of drought-related research and are the most promising sources of drought-related gene [13]. However, due to the rapid loss of genetic variation from cultivar replacement, modern barley cultivars have become more sensitive to abiotic and biotic stresses, and their monotonous genetic background has been an obstacle to breeding improved cultivars. Wild barley offers the prospect of a ‘goldmine’ of untapped genetic reserves [12]. The identification of well-adapted wild relatives that are able to grow well in drought-prone soils provides a useful supply of new germplasm for future breeding. Tibetan annual wild barley from Qinghai-Tibet Plateau is regarded as one of the progenitors of cultivated barley, and it is rich in genetic diversity [14]. Our previous study [15] successfully identified two contrasting Tibetan barley genotypes XZ5 (drought-tolerant) and XZ54 (drought-sensitive) in response to drought stress. However, the protein expression involved in response to drought stress in Tibetan wild type barley have never been investigated and compared with elite cultivars under drought stress. Thus, the question arises whether the mechanism for drought-tolerance in wild barley genotype XZ5 is associated with the related protein/gene expression. If this is the case, the question arises whether the proteins associated with drought tolerance in Tibetan wild barley are different from those in cultivated barley. This knowledge is important for understanding the mechanisms underlying tolerance to drought stresses in wild barley.

In this study, we investigated stress-specific proteins associated with drought tolerance in wild barley by comparing the proteomic responses of the two contrasting Tibetan wild barley genotypes XZ5 (high drought tolerant), XZ54 (drought sensitive) and *cv.* ZAU3 using two-dimensional gel electrophoresis (2-D) and mass spectrometry (MS). These results are useful to better understand the mechanisms of drought tolerance in barley, and provide an effective pathway for the exploration of drought-tolerant genes in plants.

## Methods

### Plant materials and experimental design

A greenhouse pot experiment was carried out on Huajiachi Campus, Zhejiang University, Hangzhou, China. Agricultural silt loam soil was collected from the experimental farm (depth 0–15 cm) in Huajiachi campus. Soil was air-dried and mixed daily until 8 % water content was reached. Air-dried soil was sieved and plastic pots (6 L, 20 cm height) were filled with 4.5 kg air-dried soil. The soil used in this investigation had a pH of 6.9, with a total N, and available P, K of 2.4 g kg^−1^, and 38.2, 31.5 mg kg^−1^, respectively.

Two contrasting Tibetan wild barley genotypes XZ5 (drought-tolerant) and XZ54 (drought-sensitive) (*H. vulgare* L. *ssp. spontaneum*) [15] and one cultivated barley *cv*. ZAU3 was used in this study. Seeds were sown in each pot, and thinned to thirteen seedlings 10 days after germination (10 DAE). Drought treatment was conducted at two-leaf stage (20 DAE). There were 2 treatments: control, in which soils in the pots were kept humid (60–80 % water holding capacity) throughout; drought stress, seedlings were subjected to drought stress for 20 days by withholding irrigation until the soil moisture content was reduced to around 4 %. After acquiring 4 % soil moisture, the treated pots were subsequently watered to re-establish a soil humid of 60-80 % water holding capacity for 5 day recovery. The experiment was arranged in a split-plot design, with treatments as the main plot and genotypes as the sub-plot with twelve replicates. Soil moisture was measured using an HH2 Moisture Meter (Delta-T Devices, Cambridge, UK) every day. Plants were sampled when the soil moisture content (SMC) was at 10 % (after 9 day treatment) and 4 % (after 20 day treatment); and after 2 day and 5 day re-watering (60–80 % water holding capacity), respectively, which were denoted by SMC10 and SMC4, and R1 and R2. Fresh leaves with three replicates of ten plants each from each genotype and condition were flash frozen in liquid nitrogen, and stored at −80 °C for protein extraction.

### Protein extraction and quantification

Total leaf protein extracts were prepared essentially according to phenol extraction method described by Bah et al. [16]. Protein concentration was determined by standard Bradford assay using bovine serum albumin as standard (Bio-Rad, Hercules, CA, USA). All chemicals used were, if not further specified in the text, p.a. or electrophoresis grade. All electrophoresis units employed were from Amersham Biosciences. For each sample, at least three independent protein extracts were prepared after each treatment and at least three 2-DE analyses were performed for each protein extract.

### Two-dimensional gel electrophoresis, protein visualization, image analysis and quantification

Protein visualization, image analysis and quantification were determined according to Bah et al. [16]. To analysis the expressed protein patterns, stained gels were scanned and calibrated using a PowerLook 1100 scanner (UMAX), followed by analysis of protein spots using GE HealthCare Software (Amersham Biosciences). The protein spots were quantified using the % volume criterion. Only those with significant and reproducible changes (*p* < 0.05) were considered to be differentially accumulated proteins. The target protein spots were automatically excised from the stained gels and digested with trypsin using a Spot Handling Workstation (Amersham Bio-sciences). Peptides gel pieces were placed into the EP tube and washed with 1:1 mixture of 50 μL of 30 mM K_3_Fe(CN)_6_ and 100 mM NaS_2_O_3_ for 10–15 min until completely discolored then washed with 200 μL bi-distilled water (two times for 5 min each). The washed solution was drained and washed with 50 % ACN (acetonitrile, Fisher A/0626/17) and 100 % ACN rotationally, and then incubated in 25 mM NH4HCO3 (Sigma A6141) for 5 min at 37 °C. After leaching out of the incubation solvent, 50 % ACN and 100 % ACN was rotationally added and dried at 40 °C for 5 min respectively. Trypsin digestion was carried out as follows: sequencing-grade porcine trypsin (Promega, Madison, WI, USA) was suspended in 25 mM NH4HCO_3_ at a concentration of 12.5 ng per ml to rehydrate the dried gel pieces. The trypsin digestion was carried out for 16 h at 37 °C. Peptides were extracted from the digest as follows for three times: 10 mL of 50 % ACN containing 0.1 % TFA (trifluoroacetic acid, GE HealthCare) was added to each tube and incubated for 5 min at 37 °C and the supernatants were transferred to new EP tube. The extracts were pooled and then vacuum concentrated for about 2 h. A solution of peptides was filtrated via Millipore (Millipore ZTC18M096) and mixed with the same volume of a matrix solution consisting of saturated a-cyano-4-hydroxycinnamic acid (CHCA) in 50 % ACN containing 0.1 % TFA. After the peptides were co-crystallized with CHCA by evaporating organic solvents, tryptic-digested peptide masses were measured using a MALDI-TOF-TOF mass spectrometer (ABI4700 System, USA). All mass spectra were recorded in positive reflector mode and generated by accumulating data from 1000 laser shots. The following threshold criteria and settings were used: detected mass range of 700–3200 Da (optimal resolution for the quality of 1500 Da), using a standard peptide mixture (des-Argl-Bradykinin Mr904.468, Angiotensin I Mr1296.685, Glul-Fihrinopeptide B Mr1570.677, ACTH (1–17) Mr2093.087, ACTH (18–39) Mr2465.199; ACTH (7–38) Mr3657.929) as an external standard calibration, with laser frequency of 50 Hz, repetition rate of 200 HZ, UV wavelength of 355 nm, and accelerated voltage of 20,000 V. Peptide mass fingerprint data were matched to the NCBInr database using Profound program under 50 ppm mass tolerance.

### Peptide and protein identification by database search

Data were processed using the Data Explorer software (Applied Biosystems) and proteins were unambiguously identified by searching against a comprehensive non-redundant sequence database (NCBInr) using the MASCOT software search engine (http://www.matrixscience.com/cgi/search_form.pl?FORMVER=2&SEARCH=MIS). Folds of increase and decrease in drought treated (drought) *vs* untreated (control) leaves were calculated as drought/control and -control/drought for up- and down-regulated proteins, respectively. For single-peptide identified proteins, up- and down-regulation were assigned when the regulation factors were above 1.5 folds (*p* < 0.05) [16].

### qRT-PCR analysis

The pot experiment was carried out again using XZ5, XZ54 and *cv*. ZAU3 under control and drought stress treatment with three replicates as described above. Total RNA was isolated from leaves of barley plants under control and SMC 10 % and 4 % using the TRIzol reagent following manufacturers’ recommendation (Invitrogen, Karlsruhe, Germany). Residual DNA was removed using purifying columns. One microgram of each RNA sample was subsequently employed for cDNA synthesis with 0.5 μg of oligo (dT) 12-18 and 200 units of Superscript II (Invitrogen, Karlsruhe, Germany). cDNA samples were assayed by quantitative real time PCR (qRT-PCR) in the iCycler iQTM Real-time PCR Detection System (Bio-Rad, Hercules, CA, USA) using the SYBR Green PCR Master Mix (Applied Biosystems). The PCR conditions consisted of denaturation at 95 °C for 30 s, followed by 40 cycles of denaturation at 95 °C for 5 s and annealing at 60 °C for 30 s. Gene-specific primers (Additional file 1: Table S1) were designed using the Primer Express software (Applied Biosystems). Barley *GAPDH* (glyceraldehyde-3-phosphate dehydrogenase) gene (accession no. M36650, fw-5’-AAGCATGAAGATACAGGGAGTGTG-3’, rv-5’-AAATTTATTCTCGGAAGAGGTTGTACA-3’) and barley *ACTIN* (AY145451, fw-5’-ATGTTTTTTTCCAGACG-3’, rv- 5’-ATCAAGCCAACCCAAGT-3) were used as control.

### Cloning the coding sequences (CDS) of *rbcL*and *Trx-M* genes from XZ5, XZ54 and ZAU3

The two chloroplast genes analyzed were Ribulose-1,5-bisphosphate carboxylase/oxygenase large subunit (*rbcL*) and Thioredoxin M, chloroplast precursor (*Trx-M*). Total RNA was extracted from the leaves of XZ5, XZ54, and ZAU3, using RNAprep Pure Plant Kit (TIANGEN BIOTECH, ID: DP432) according to the manufacturer’s instructions. The following primers were used to amplify *rbcL* and *Trx-M*, respectively: rbcL1 (TAGACCCTGTTATTGTGAGA, 5’ to 3’) and rbcL2 (GAATTTGATCGCCTTCC, 3’ to 5’), Trx-M1 (GCAATGGCCTTGGAGA, 5’ to 3’) and Trx-M 2 (GCTGCCGATGTACTTGTC, 3’ to 5’). Two pairs of primers were designed from the published sequences of *Hordeum vulgare* L *rbcL* and *Trx-M* gene (GenBank Accession Numbers: AY137456.1 and AK360709.1. The purified RNA samples were reverse-transcribed using HiScriptTM 1st strand cDNA Synthesis Kit (Vazyme biotech Co., Ltd.). The following reaction mixture was prepared for all amplifications: 10 × *Ex Taq* buffer (Mg^2+^ Plus), 0.3 μM each primer, 0.2 mM each dNTP Mixture, 70 ng DNA template, 1 unit of high-fidelity *Ex Taq* DNA polymerase (TaKaRa Biotechnology, ID: RR001A), and Sterilized distilled water to a final volume of 25 μl. For amplification of chloroplast genes, PCR was conducted using the following protocol: 1 min denaturation at 94 °C; followed by 30 cycles of 5 s at 98 °C, 30 s at different temperature (according to genotypes, see Additional file 2: Table S2), 2 min at 72 °C; and a final extension of 10 min at 72 °C. PCR products of the two genes were screened on 1 % agarose gels and purified using the Universal DNA Purification Kit (TIANGEN BIOTECH, ID: DP214). Purified PRC products were subsequently cloned into a pMDTM-19 T vector (TaKaRa) according to the manufacturer’s instructions, and the positive clones were randomly selected and sequenced by Sangon Biotech (Shanghai) Co., Ltd.

## Results

### 2-DE analysis of leaf proteins in the three genotypes under drought and control condition

**Fig. 1:**
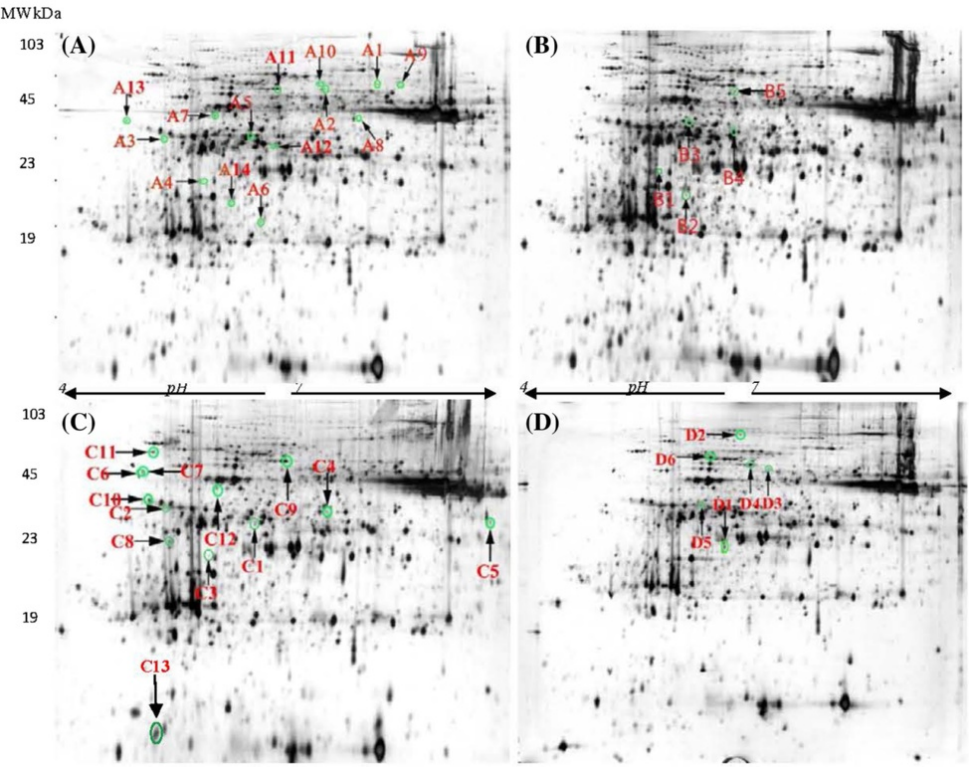
Representative two-dimensioal gel electrophpresis maps of leaf proteins of XZ5 after different days of drought stress. The proteins were isolated from the leaves of XZ5 plants exposed to drought for 9 days (soil moisture content, SMC 10 %, **a)**, 20 days (SMC 4 %, **b)**, and after 2 day re-watering (60-80 % water holding capacity, **c)** and 5 day re-watering **(d)**, respectively. Total proteins were extracted and separated by 2-DE. In IEF, 100 mg proteins were loaded onto pH 4–7 IPG strips (24 cm, linear). SDS-PAGE was performed with 12.5 % gels. The spots were visualized by silver staining. Differentially accumulated protein spots are indicated by green sashes, and marked with arrows and numbers

Protein spots were visualized by silver blue-staining. Fig. 1 and (Additional file 3: Figure S1; Additional file 4: Figure S2; Additional file 5: Figure S3). show the entire image of 2-DE gels (isoelectric focusing pH range, 4–7; size, 24 cm) of total extracted leaf proteins from the 3 barley genotypes under drought and control condition*.* On average of the 4 sampling data, total leaf proteins of control plants were resolved into 2312, 2123 and 2172 spots, in XZ5, XZ54, and ZAU3, respectively, in each reproducible 2-DE gels (Additional file 3: Figure S1, Additional file 4: Figure S2A-D and S3A-D). The averages of protein spots of 2-DE gels in drought stressed plants were 2419 (SMC10, soil moisture content at 10 %), 2310 (SMC4, SMC at 4 %), 2340 (R1, after 2 days of recovery following drought stress), 2343 (R2, 5 days of recovery) in XZ5 (Fig. 1), 2265, 2341, 2182, 2217 in XZ54 (Additional file 4: Figure S2E-H), and 2154, 2254, 2308, 2192 in ZAU3 (Additional file 5: Figure S3E-H), respectively. Comparing 2-DE gels from control and from drought stressed samples showed many differences in protein presence. A 1.5-fold quantitative change was set as the criteria. Overall, 132 (SMC10), 92 (SMC4), 86 (R1), 242 (R2) protein spots were found to be altered by drought stress in XZ5, respectively, with 75, 64, 42, 53 up-regulated by drought. Concerning XZ54 and ZAU3, protein spots altered by drought stress were 261, 137, 155, 187 and 122, 116, 126, 176, respectively, with 212, 103, 121, 137 and 45, 79, 79, 109 up-regulated by drought.

### Differential drought-induced protein expression in leaves of the three genotypes

**Fig. 2:**
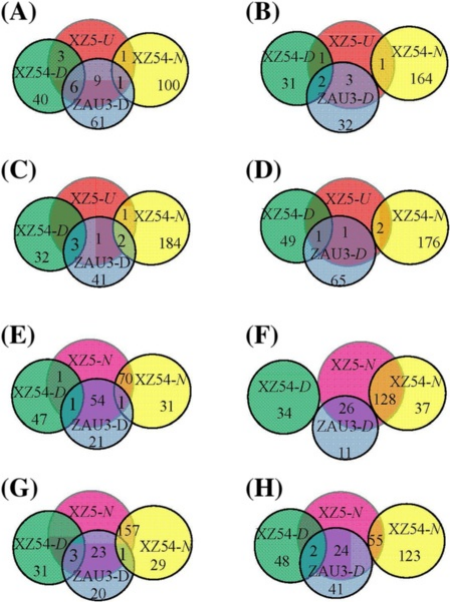
Venn diagram illustrating the expression patterns of drought stress-responsive proteins in leaves of XZ5, XZ54 and ZAU3. The numbers of differentially expressed spots up-regulated or non-change in XZ5 are shown in the different segments. The proteins were isolated from the leaves of XZ5, XZ54 and ZAU3 plants exposed to drought for 9 day (soil moisture content, SMC 10 %, **a** and **e)**, 20 day (SMC 4 %, **b** and **f)**, and after 2 day re-watering (60-80 % water holding capacity, **c** and **g)** and 5 day re-watering **(d** and **h)**, respectively. *U*, up-regulated; *D*, down-regulated; *N*, non-change under drought stress

**Fig. 3:**
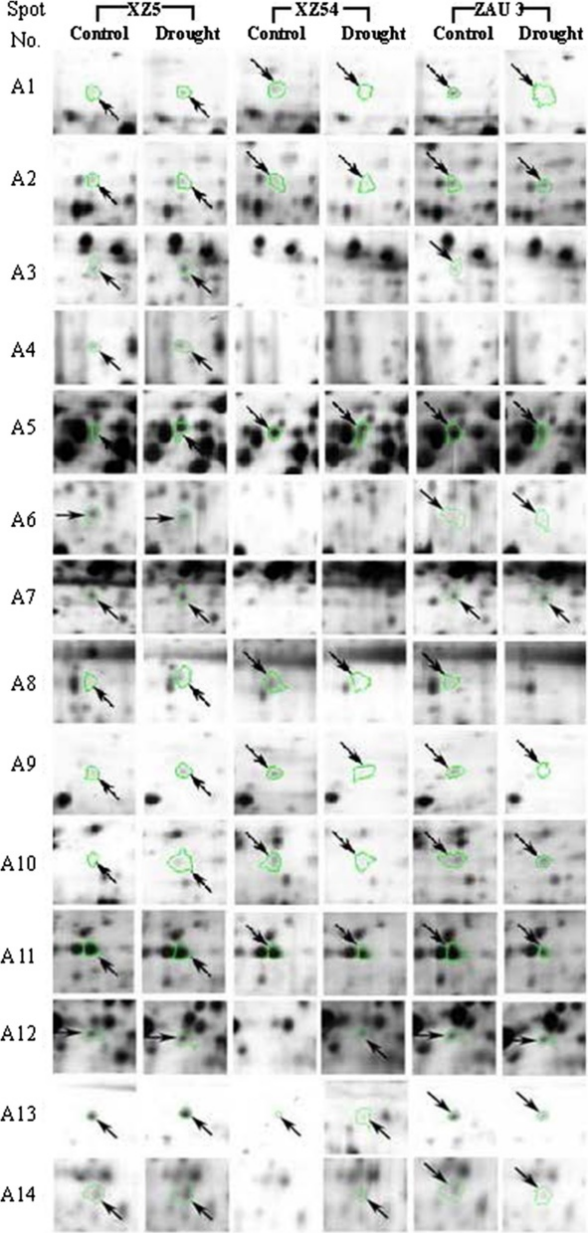
‘Spot view’ of the abundance of differentially expressed proteins (indicated with green circles) in leaves of three barley genotypes XZ5, XZ54 and ZAU3 under control and drought stress (SMC 10 %). Protein spot ID refers to numbers in Fig. 1a and Tables 1 and 3

**Fig. 4:**
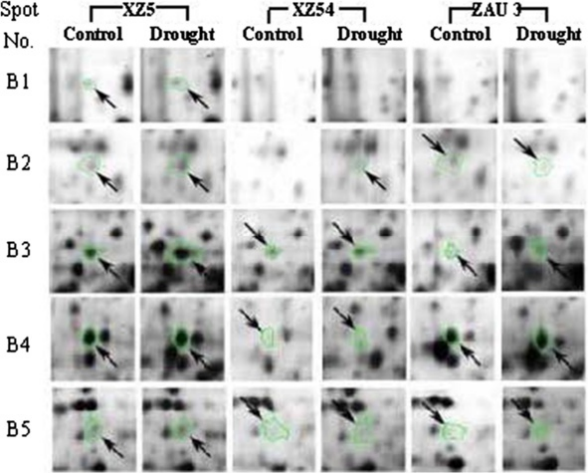
‘Spot view’ of the abundance of differentially expressed proteins (indicated with green circles) in leaves of three barley genotypes XZ5, XZ54 and ZAU3 under control and drought stress (SMC 4 %). Protein spot ID refers to numbers in Fig. 1b and Tables 2 and 3

Further comparison of the genotypic differences in drought altered protein spots, we found 59 (at SMC10), 51 (SMC4), 38 (R1), 107 (R2) drought-responsive protein spots, respectively (Fig. 2). Among them, 29 spots, whose expression was significantly induced in XZ5 leaves but down-regulated/unchanged in XZ54, or unchanged in XZ5 but down-regulated in XZ54, were excised and analyzed by matrix assisted laser desorption ionization time-of-flight mass spectrometry (MALDI-TOF/TOF MS). These 29 spots included 11 (at SMC10, spots A1-A11), 3 (SMC4, spots B1-B3), 10 (R1, spots C1-C13), 5 (R2, spots D1-D5) spots. Some excised proteins were unambiguously identified by MS and data bank analysis *via* matching to proteins from *H. vulgare* and homologous proteins of other green plants in the NCBI non-redundant (nr) protein database and barley ESTs databases (Figs. 1, 3 and 4; Additional file 3: Figure S1, Additional file 4: Figure S2, Additional file 5: Figure S3, Additional file 6: Figure S4; Tables 1 and 2).

**Table 1:** Proteins whose expression was significantly induced (+) in XZ5 leaves but down-regulated (-) /unchanged in XZ54, or unchanged in XZ5 but down-regulated in XZ45 after 9 and 20 day drought stress

Spot No.	Protein name	C. I. (%)	Accession number	MW (Da)	pI	AASC (%)	Fold increase (+) or decrease (-)			Function
							XZ5	XZ54	ZAU3	
	Nine day drought stress (SMC 10 %)									
A1	Ribulosebisphosphate carboxylase small chain clone 512 [*Triticum aestivum*]	100	gi|132107	13275	5.8	35.0	+2.8	−2.2	−0.2	Photosynthesis
A2	Adenosylhomocysteinase [*Nicotiana tabacum*]	99.9	P68173|SAHH_TOBAC	53070	5.5	23.3	+1.9	−2.4	−1.9	Amino-acid biosynthesis
A3 (C2)^a^	Melanoma-associated antigen p97 [*Gallus gallus*]	96.2	gi|45383930	80860	5.9	22.0	+1.7	NE	−10^6^	Stress response
A4 (B1 C3)	Type I chlorophyll a/b-binding protein b [*Amaranthus tricolor*]	100	gi|13676406	16608	4.6	18.8	+5.6	NE	NE	Photosynthesis
A5	Unnamed protein product	100	gi|74190672	41738	5.4	37.6	+2.8	−0.1	+0.003	Unknown
A6	Glutathione S-transferase 1 [*T. aestivum*]	100	P30110|GSTF1_WHEAT	25811	5.3	29.3	+0.1	NE	−1.7	Stress response
A7	Ribulosebisphosphate carboxylase large chain (RuBisCO large subunit) [*Welwitschia mirabilis*]	99.2	RBL_WELMI	49579	6.3	25.1	+0.5	NE	+2.2	Photosynthesis
A8	Ribulosebisphosphate carboxylase large chain Precursor [*Saccharum hybrid*]	99.9	Q6L391|RBL_SACHY	52695	6.3	21.6	+4.5	−2.1	−10^6^	Photosynthesis
A9	Ribulosebisphosphate carboxylase large chain precursor [*T. aestivum*]	100	RBL_WHEAT	53445	6.2	34.8	+3.0	+0.1	−3.0	Photosynthesis
A10 (D3)	Ribulosebisphosphate carboxylase large chain precursor [*Pisum sativum*]	98.8	P04717|RBL_PEA	52730	6.6	25.5	+11.4	−2.6	−1.8	Photosynthesis
A11 (C9 D4)	FTSH1 (FtsH protease 1); ATP-dependent peptidase/ATPase/ metallopeptidase [*Arabidopsis thaliana*]	100	gi|18402995	76712	5.6	19.0	+1.3	−2.5	1.4	Stress response
	Twenty day drought stress (SMC 4 %)									
B1 (A4 C3)	Type I chlorophyll a/b-binding protein b [*Amaranthus tricolor*]	100	gi|13676406	16608	4.6	18.8	+10^6^	NE	NE	Photosynthesis
B2 (A14)	Transketolase, chloroplast [*Zea mays*]	99.9	Q7SIC9|TKTC_MAIZE	72948	5.5	15.1	+1.5	NE	NE	Photosynthesis
B3	ATP synthase beta subunit [*Catabrosa aquatica*]	100	gi|110915610	49371	5.1	53.5	+2.5	−2.1	+2.8	Energy

AASC, Amino acid sequence coverage; Protein spot ID refers to numbers in Fig. 1a-b. Accession number of top database match from the NCBInr database. Fold increase and decrease were calculated as drought/control, and –control/drought for up and down -regulated proteins, respectively. All ratios shown are statistically significant (*p* < 0.05). +10^6^ and -10^6^ referred to the specific expressed and totally inhibited proteins, respectively. NE, Non Expression. SMC, soil moisture content. ^a^the same protein spots identified at different stages

**Table 2:** Proteins whose expression was significantly induced (+) in XZ5 leaves but down-regulated (-) /unchanged in XZ54, or unchanged in XZ5 but down-regulated in XZ54 after 2 and 5 days of recovery following drought stress

Spot No.	Protein name	C. I. (%)	Accession number	MW (Da)	pI	AASC (%)	Fold increase (+) or decrease (-)			Function
							XZ5	XZ54	ZAU3	
	Two days of recovery									
C1	Ribulose bisphosphate carboxylase/oxygenase activase, chloroplast precursor [*Hordeum vulgare*]	100	Q40073|RCAA_HORU	51041	8.0	41.8	+1.6	−1.7	−1.9	Photosynthesis
C2	Melanoma-associated antigen p97 [*G. gallus*]	96.2	gi|45383930	80860	5.9	22.0	+10^6^	NE	NE	Stress response
(A3)^a^										
C3	Type I chlorophyll a/b-binding protein b [*A. tricolor*]	100	gi|13676406	16608	4.6	18.8	+10^6^	NE	NE	Photosynthesis
(A4 B1)										
C4	Glyceraldehyde-3-phosphate dehydrogenase 2 [*A. thaliana*]	100	gi|186478427	37644	7.6	14.6	+3.3	−1.2	−10^6^	Metabolism
C5	Os02g0739600 Putative pyruvate dehydrogenase E1 alpha subunit [*Oryza sativa*]	100	gi|115448577	42675	7.6	34.6	+3.0	−1.3	−1.4	Metabolism
C6	Elongation factor G [*A. thaliana*]	100	gi|62320532	47645	5.4	21.4	+1.2	−1.7	−1.7	Amino-acid biosynthesis
C7	Elongation factor G [*A. thaliana*]	100	gi|62320532	47645	5.4	21.4	+1.2	−1.7	−1.7	Amino-acid biosynthesis
C8	Putative ascorbate peroxidase [*T. aestivum*]	100	gi|25992559	39922	5.5	5.5	+5.5	+1.2	−1.9	Stress response
C9	FTSH1 (FtsH protease 1); ATP-dependent peptidase/ATPase/ metallopeptidase [*A. thaliana*]	100	gi|18402995	76712	5.6	19.0	−0.02	−1.7	−3.9	Stress response
(A11 D4)										
C13	Thioredoxin M-type, chloroplast precursor (Trx-M)	100.0	gi|11135474	19120	8.7	44.0	+1.9	−1.6	−1.1	Stress response
	Five days of recovery									
D1	Hypothetical protein [*Sporobolus stapfianus*]	99.9	gi|1808684	31866	5.7	21.8	+10^6^	+1.3	+1.4	Unknown
D2	Os05g0405000 [*O. sativa*]	100	gi|115463815	102722	6.0	18.5	+1.8	−2.7	−5.7	Photosynthesis
D3	Ribulosebisphosphate carboxylase large chain precursor [*P. sativum*]	98.8	P04717|RBL_PEA	52730	6.6	25.5	−0.03	−1.5	−2.2	Photosynthesis
(A10)										
D4	FTSH1 (FtsH protease 1); ATP-dependent peptidase/ATPase/ metallopeptidase [*A. thaliana*]	100	gi|18402995	76712	5.6	19.0	−1.4	−2.3	−6.3	Stress response
(A11 C9)										
D5	ATP synthase CF1 beta subunit [*H. vulgare*]	100	gi|118430395	53841	5.2	52.8	+2.6	−0.08	+1.5	Energy

AASC, Amino acid sequence coverage; Protein spot ID refers to numbers in Fig. 1c-d. Accession number of top database match from the NCBInr database. Fold increase and decrease were calculated as drought/control, and –control/drought for up and down -regulated proteins respectively. All ratios shown are statistically significant (p < 0.05). +10000 and -10000 referred to the specific expressed and totally inhibited proteins, respectively. NE, non expression. ^a^ the same protein spots identified at different stages

As to the proteins changed in a similar manner in the four sampling data (SMC10, SMC4, R1 and R2) of XZ5, as shown in Tables 1, 2 and 3, drought induced the expression of ribulose bisphosphate carboxylase, type I chlorophyll a/b-binding protein b, ATP-dependent peptidase/ATPase/ metallopeptidase at three different sampling stages. Two proteins ATP synthase beta subunit and mono-dehydroascorbate reductase changed similar in both of SMC4 and R2 samples of XZ5 under drought stress. And melanoma-associated antigen p97, transketolase and elongation factor G changed similar in both of SMC10 and R1, SMC10 and SMC4, SMC4 and R1 samples, respectively of XZ5 under drought stress (Tables 1, 2 and 3).

**Table 3:** Proteins whose expression were significantly higher expressed (+) in XZ5 compared with XZ54 leaves under control condition (XZ5 *vs* XZ54)AASC, Amino acid sequence coverage; Protein spot ID refers to numbers in Fig. 1. Accession number of top database match from the NCBInr database. Fold increase and decrease were calculated as drought/control, and –control/drought for up and down -regulated proteins respectively. All ratios shown are statistically significant (*p* < 0.05). +10^6^ and -10^6^ referred to the specific expressed and totally inhibited proteins, respectively

Spot No.	Protein name	C. I. (%)	Accession number	MW (Da)	pI	AASC (%)	Fold increase (+) or decrease (-)				
							XZ5 *vs* XZ54^a^	XZ5^b^	XZ54	ZAU3	Function
	Nine day drought stress (SMC 10 %)										
A3	Melanoma-associated antigen p97 [*G. gallus*]	96.2	gi|45383930	80860	5.9	22.0	+10^6^	1.7	NE	−10^6^	Stress response
(C2)^c^											
A4	Type I chlorophyll a/b-binding protein b [*A. tricolor*]	100	gi|13676406	16608	4.6	18.8	+10^6^	5.6	NE	NE	Photosynthesis
(B1C3)											
A6	Glutathione S-transferase 1 [*T. aestivum*]	100	P30110|GSTF1_WHEAT	25811	5.3	29.3	+10^6^	+0.6	NE	−1.7	Stress response
A7	Ribulosebisphosphate carboxylase large chain (RuBisCO large subunit) [*W. mirabilis*]	99.2	RBL_WELMI	49579	6.3	25.1	+10^6^	+0.5	NE	+2.2	Photosynthesis
A12	Ribulosebisphosphate carboxylase/oxygenaseactivase B (RuBisCOactivase B) [*A. thaliana*]	99.4	gi|10720253	47199	7.6	24.9	+10^6^	−0.1	+10^6^	−1.2	Photosynthesis
A13	Putative ankyrin repeat domain protein 2 [*O. sativa*]	100.0	gi|108712139	37253	4.6	10.0	+29.6	+1.4	+0.1	−1.9	Metabolism
A14	Transketolase,chloroplast [*Z. mays*]	99.9	Q7SIC9|TKTC_MAIZE	72948	5.5	15.1	+10^6^	+1.6	+10^6^	−2.3	Photosynthesis
(B2)											
	Twenty day drought stress (SMC 4 %)										
B4	Putative mono-dehydroascorbate reductase, Os09g0567300 [*O. sativa*]	100	gi|115480733	46644	5.5	17.9	+7.3	+1.2	+2.3	+1.1	Stress response
B5	Elongation factor G [*A. thaliana*]]	100	gi|62320532	47645	5.45	21.4	+1.8	−0.03	−1.4	−0.5	Amino-acid biosynthesis
	Two days of recovery										
C10	Putative ascorbate peroxidase [*T. aestivum*]	100	gi|25992559	39922	5.5	5.5	+1.6	+1.8	+1.8	−1.3	Stress response
C11	Heat-shock protein [*Secale cereale*]	100	gi|556673	88063	4.9	8.2	+2.3	+0.1	−0.1	−2.5	Stress response
C12	Ribulose bisphosphate carboxylase large chain (RuBisCO large subunit) [*W. mirabilis*]	99.2	RBL_WELMI	49579	6.3	25.1	+10^6^	−0.1	+10^6^	+1.7	Photosynthesis
	Five days of recovery										
D6	Putative mono-dehydroascorbate reductase, Os09g0567300 [*O. sativa*]	100	gi|115480733	46644	5.5	17.9	+3.2	+0.2	−0.1	+1.4	Stress response

^a^ Fold increase under control condition in XZ5 *vs* XZ54

^b^ Fold increase or decrease in XZ5, XZ54 and ZAU3 (drought *vs* control)

^c^ the same protein spots identified at different stages

In addition, expression of 13 proteins (Spots: A3, A4, A6, A7, A12, A13, A14, B4, B5, C10-C12, D6), classified as the 4 category (Table 3) was significantly higher in XZ5 *vs* XZ54 under control condition, and simultaneously induced or non-changed under drought in XZ5. These proteins may be also potentially responsible for the drought tolerance in XZ5 compared with ZAU3.

Among the 38 identified spots, 7 (A3, A4, A6, A7, A12, A14, C12; Table 3) proteins expressed in XZ5 but not expressed in XZ54. Further comparison of the 38 identified spots with that of ZAU3 revealed that 21 proteins up-regulated in XZ5 were surprisingly down-regulated or unaltered in both ZAU3 and XZ54; 2 proteins (A7 and B3) were slightly up-regulated in ZAU3 under drought. There were 5 protein spots uniquely expressed (A4, B1 and C3 were type I chlorophyll a/b-binding protein b; B2 was transketolase, chloroplast; C2 was melanoma-associated antigen p97) in XZ5 under drought (Tables 1 and 2).

### Functional classification of the drought stress responsive proteins

**Fig. 5:**
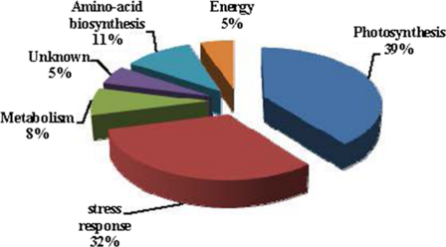
The functional category distribution of the 38 identified proteins in barley leaves subjected to drought

These 38 proteins were classified into 6 groups based on their bio-chemical functions. The majority of the protein profile was photosynthesis (spots A1, A4, A7, A8, A9, A10, A12, A14, B1, B2, C1, C3, C12, D2, D3), stress response (A3, A6, A11, B4, C2, C8, C9, C10, C11, C13 D4, D6), amino-acid biosynthesis (A2, B5, C6, C7) and metabolism process (A13, C4, C5). The other two minor groups included energy (B3, D5) and unknown (A5, D1) (Fig. 5, Tables 1, 2, and 3).

### XZ5 shows higher expression of genes corresponding to drought up-regulated proteins

**Fig. 6:**
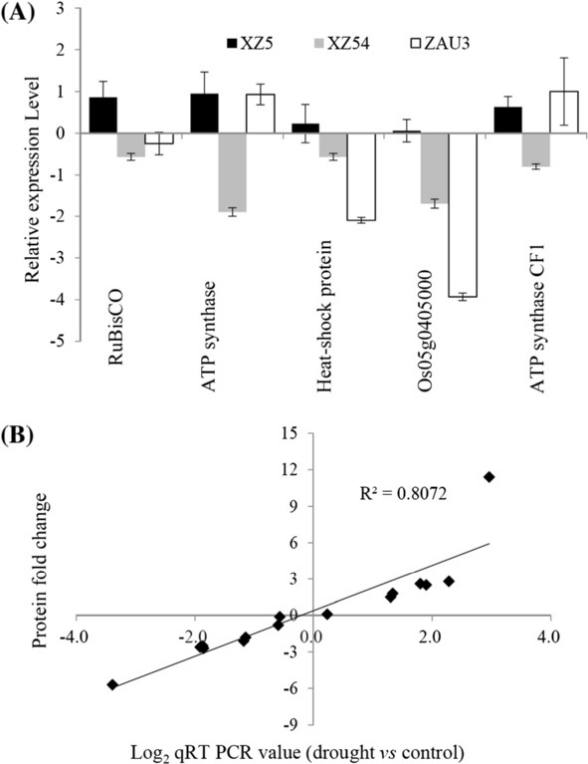
Quantitative Real-Time PCR analysis of the five drought inducible proteins **a**, and correlation of qRT-PCR (log_2_ scale) and protein fold change **b**. Barley *GAPDH* and *ACTIN* were used as the reference genes. Data plotted are the mean ratio of gene/protein expression on the four sampling dates (SMC10, SMC4, R1 and R2) in drought treatment over those in the control in the XZ5, XZ54 and ZAU3 on a log_2_ scale (*R* ^2^ = 0.8072, *P* < 0.01). SMC10 and SMC4, and R1 and R2 represent soil moisture content at 10 % and at 4 %, respectively, and after 2 and 5 days of recovery following drought stress

To determine whether the changes in protein abundance detected by 2-DE were correlated with changes at the transcriptome level, quantitative RT-PCR was performed using RNA isolated from the leaves of a separate set of plants treated with four periods (soil moisture content 10 % (SMC10) and 4 % (SMC10) and subsequently 2 days (R1) and 5 days (R2) of recovery). Transcript levels of five drought inducible proteins including ribulosebisphosphate carboxylase large chain precursor, ATP synthase beta subunit, heat-shock protein, Os05g0405000 and ATP synthase CF1 beta subunit (A10, B3, C11, D2 and D5) were chosen and successfully detected. Among them, ribulosebisphosphate carboxylase large chain precursor, heat-shock protein and Os05g0405000 were up-regulated in XZ5 but down-regulated in XZ54 and ZAU3. ATP synthase beta subunit and ATP synthase CF1 beta subunit were up-regulated in XZ5 and ZAU3 but down-regulated in XZ54, following the expression trend detected by 2-DE (Fig. 6; Tables 1, 2, and 3).

### Comparing the CDS sequence of *rbcL* and *Trx-M* genes from XZ5, XZ54 and ZAU3

The protein Ribulosebisphosphate carboxylase large chain precursor (Spot A10) and Thioredoxin M-type, chloroplast precursor (Trx-M) (Spot C13) were obtained by the results of 2-DE, and the corresponding genes could also be found in GenBank (Accession no. AY137456.1 and AK360709.1). The CDS length of *rbcL* and *Trx-M* genes was 1401 bp (the partial-length CDS region) and 528 bp (the full-length CDS region) (Additional file 7: Figure S5; Additional file 8: Figure S6), respectively, encoding 467 and 176 amino acids, respectively, and both contained no introns. Conserved domain prediction by SMART (http://smart.embl-heidelberg.de/) indicates that *rbcL* have two conserved domains: RuBisco_large_N domain and RuBisco_large_domain, their functions are ribulose-bisphosphate carboxylase activity and magnesium ion binding. For RuBisco_large_N domain the interval from XZ5, XZ54 and ZAU3 was 21-146, for RuBisco_large_domain the intervals from XZ5, XZ54 and ZAU3 were 154-462, 154-433, 154-462, respectively. And 69-172 conserved Thioredoxin domain interval belonged to *Trx-M* from XZ5, XZ54 and ZAU3, their function is cell redox homeostasis. The CDS and the amino acid sequence of the putative protein are shown in Additional files 7 and 8. Nucleotide and amino acids sequence alignment of *rbcL* and *Trx-M* from XZ5, XZ54 and ZAU3, showed that *rbcL* gene sequence contained 5 novel single nucleotide polymorphism sites (SNPs) at position 114 bp, 523 bp, 812 bp, 837 bp, 1017 bp (Table 4), respectively, of which two were missense mutations, and two missense mutation occurred in the conserved domain according to the result of sequence alignment by NCBI. Moreover, *Trx-M* gene CDS contained 3 SNPs which located in 167 bp, 385 bp and 499 bp (Table 4), 3 SNPs were all missense mutations and all occurred in the conserved domain (Table 5). Comparison of the deduced amino acid sequence of *rbc*L from XZ5, XZ54 and ZAU3, indicated two amino acid residues difference between XZ5 and XZ54. Similarly, three amino acid residues difference among XZ5, XZ54 and ZAU3. This might give rise to differences in the protein structure, thereby affected its function [17], and lead to differences in drought tolerance capacity. Furthermore, SNP mutations causing protein-coding changes or gene expression alterations both have the potential to account for agronomic traits [18].

**Table 4:** The SNPs of rbcL and Trx-M among the three genotypes XZ5, XZ54 and ZAU3

Region	Gene Name	Genotypes	SNPs				
CDS	*rbcL*	Position	114	523	812	837	1017
		XZ5	A	A	C	T	C
		XZ54	G	G	T	C	T
		ZAU3	A	A	C	T	C
	*Trx-M*	Position	167	385	499		
		XZ5	C	C	A		
		XZ54	C	T	G		
		ZAU3	G	T	A

**Table 5:** Amino acid difference of rbcL and Trx-M among XZ5, XZ54 and ZAU3

Gene Name	Amino acid sequence	Genotypes		
		XZ5	XZ54	ZAU3
*rbcL*	175	Lysine (K)	Glutamic acid (E)	Lysine (K)
	271	Threonine (T)	Isoleucine (I)	Threonine (T)
	56	Proline (P)	Proline (P)	Glycine (G)
*Trx-M*	129	Arginine (R)	Cystine (C)	Cystine (C)
	167	Threomine (T)	Alanine (A)	Threomine (T)

### Analysis of sequence homology of the *rbcL* and *Trx-M* genes

**Fig. 7:**
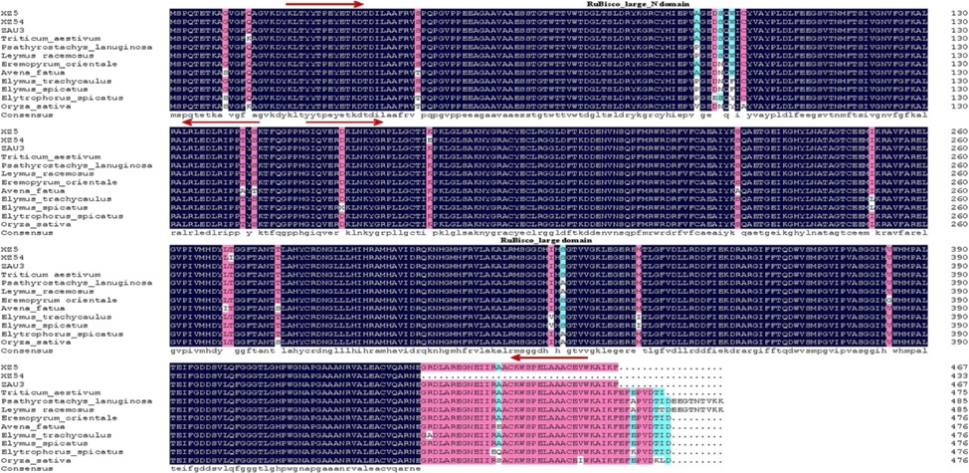
Alignment analysis of amino acid sequences of rbcL in the three barley genotypes XZ5, XZ54 and ZAU3. The dark blue represents 100 % identity; the pink represents >75 % identity; the blue-green represents 50 % identity, as defined by ClustalX (Thompson et al. 1997). The red arrow marks conserved domains

**Fig. 8:**
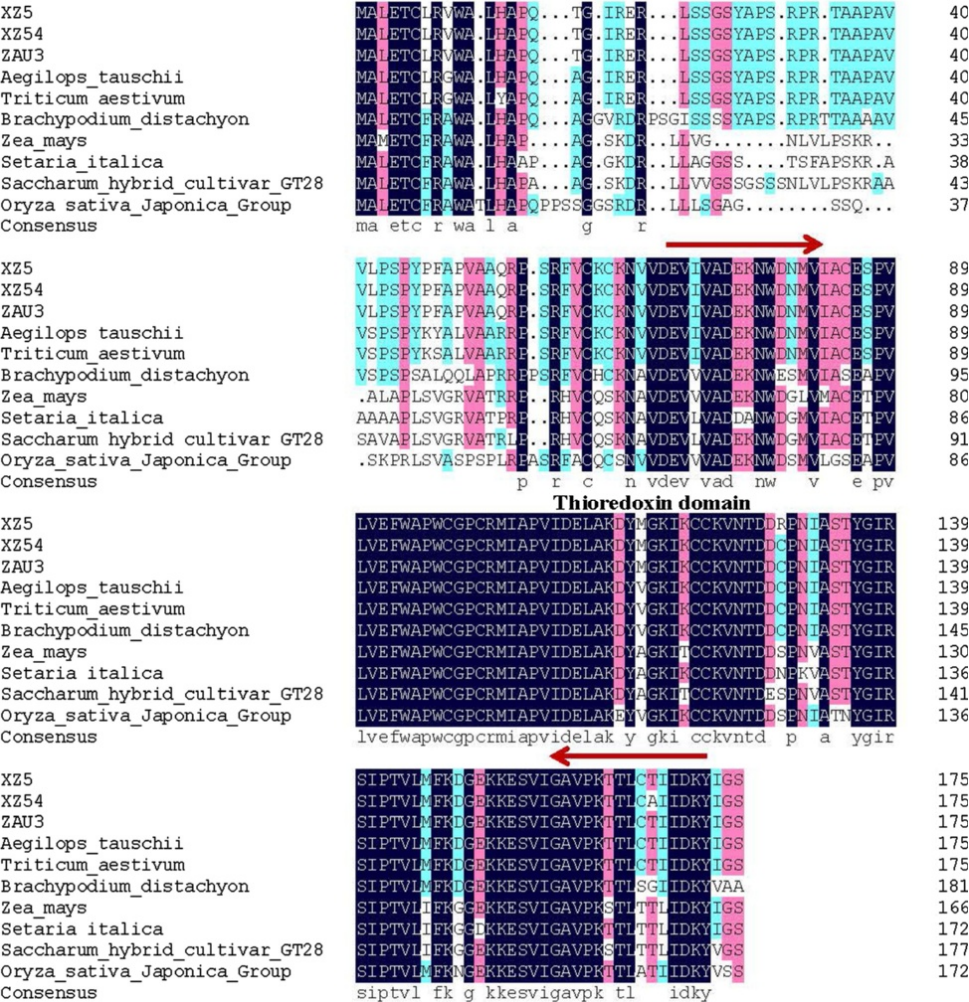
Alignment analysis of amino acid sequences of Trx-M the three barley genotypes XZ5, XZ54 and ZAU3. The dark blue represents 100 % identity; The pink represents >75 % identity; The blue-green represents 50 % identity, as defined by ClustalX (Thompson et al. 1997). The red arrow marks conserved domains

The purpose was to examine sequence homologies and divergences, nature and location of the amino acid substitutions, deletions/insertions within and between the different plants. Therefore, BLASTP searches of the *rbc*L and *Trx-M* amino acid sequences obtained in this study were performed to determine homology between the sequences with those previously reported, and revealed that *rbcL* from XZ5, XZ54 and ZAU3 aligned with that from *Triticum aestivum* (GenBank accession number: AHI44627.1), *Psathyrostachys lanuginose* (GenBank accession number: AAU11108.1), *Leymus racemosus* (GenBank accession number: ACF57874.1), *Eremopyrum orientale* (GenBank accession number: ACI95759.1), *Avena fatua* (GenBank accession number: CAG34134.1), *Elymus trachycaulus* (GenBank accession number: CAA90000.1), *Elymus spicatus* (GenBank accession number: CAA90007.1), *Oryza sativa* (GenBank accession number: CAG34174.1), *Elytrophorus spicatus* (GenBank accession number: YP_009073022.1) (94.31 % homology). Trx-M amino acid sequences from XZ5, XZ54 and ZAU3 aligned with that from *Aegilops tauschii* (GenBank accession number: EMT27162.1), *Triticum aestivum* (GenBank accession number: Q9ZP21.1), *Brachypodium distachyon* (GenBank accession number: XP_003578817.1), *Zea mays* (GenBank accession number: NP_001105330.1), *Setaria italic* (GenBank accession number: XP_004977183.1), *Saccharum hybrid cultivar* GT28 (GenBank accession number: AFO59575.1), *Oryza sativa* Japonica Group (GenBank accession number: NP_001176826.1) and *Glycine max* (GenBank accession number: NP_001237660.1) (80.86 % homology). The results of sequence alignment showed that the amino acid sequence from XZ5, XZ54 and ZAU3 had high homology with that of other plants (Figs. 7 and 8)

.

## Discussion

In the present work, drought tolerant Tibetan wild barley XZ5 recorded significantly less reduction in shoot dry weight (Additional file 9: Figure S7), when compared with control, than that of XZ54 and ZAU3 in which drought stress symptom of wilt and lodging appeared rapidly and severely, the trend was the same as that of our previous study [15]. Analysis of the proteome complement is required for a thorough understanding of the cellular processes that are associated with drought. Our current data is the first study to identify drought-responsive proteins in Tibetan wild barley (XZ5) using a proteomic approach. The leaf proteomic study identified 38 protein spots associated with drought-tolerance in wild barley XZ5 (Fig. 1; Additional file 3: Figure S1, Additional file 4: Figure S2, Additional file 5: Figure S3; Tables 1, 2, and 3), which may be specific proteins with important roles in drought tolerance in XZ5. These proteins play a role in photosynthesis, biosynthesis, energy metabolism, and unknown functions. Among them, 23 protein spots were exclusively expressed or up-regulated by drought stress in XZ5 but not XZ54, including melanoma-associated antigen p97, type I chlorophyll a/b-binding protein b, ATP synthase CF1 beta subunit, ribulosebisphosphate carboxylase large chain; of which type I chlorophyll a/b-binding protein b (spots A4, B1, C3), chloroplast transketolase (spot B2) and melanoma-associated antigen p97 (spot C2) specifically expressed in XZ5 but not XZ54 and ZAU3. Further investigations of these proteins may elucidate the mechanism of drought tolerance in XZ5 and will provide new molecular resources to develop more drought-tolerant crops. The selected stress-responsive proteins are discussed below according to their function.

### Photosynthesis related proteins

A large proportion of the proteins whose abundance changed significantly under drought are associated with photosynthesis. In the leaf quantitative proteomic analyses, 15 of the identified proteins are involved in photosynthesis: ribulosebisphosphate carboxylase large chain (RuBisCO large subunit; spots A1, A7-A10, A12, C1, C12, D3), type I chlorophyll a/b-binding protein b (spots A4, B1, C3), Os05g0405000 (spot D2) and chloroplast transketolase (spots A14 and B2) (Tables 1, 2, and 3). All Rubisco detected in this study were up-regulated in response to the drought stress in the drought tolerant wild genotype (XZ5), but down-regulated/not expressed in the sensitive wild genotype XZ54 (Tables 1-3). Rubisco, also called Fraction-I protein, accounts for up to 30–70/100 g of soluble leaf proteins (SLP) and plays a part in photosynthesis. Ribulose 1,5-bisphosphate carboxylase/oxygenase (EC 4.1.1.39, RuBisCO) catalyzes the initial step of carbon metabolism, the fixation of carbon dioxide, in photosynthetic eukaryotes. Rubisco is an extremely slow catalyst and moreover its carboxylation activity is compromised by competing side-reactions, the most notable with another atmospheric gas, O_2_, which attacks the same enediol intermediate of RuBP. The opposing oxygenase activity of Rubisco results in the synthesis of phosphoglycolate, a molecule of limited use to most organisms. Phosphoglycolate is re-circulated by photorespiration, an energy-requiring salvage pathway. This causes a constant drain on the pool of the sugar substrate (RuBP) and results in a decrease of the efficiency of carbon fixation by up to 50 %. Thus, the key to the efficiency of any particular Rubisco enzyme should be to enhance it with the ultimate aim to suppress oxygenation and improve carboxylation by Rubisco as a means to improve plant height, biomass and yield as the required characteristics of a possible candidate for drought tolerant crop genotype. Our previous studies consistent with this results that XZ5 (drought-tolerant) recorded higher photosynthesis, biomass and yield than that in XZ54 (drought-sensitive) under drought stress [15].

In addition, type I chlorophyll a/b-binding protein b specifically expressed in XZ5 but not XZ54 and ZAU3 at 10 % soil moisture content (spot A4, Table 1). Chlorophyll a/b-binding proteins acting as a mobile pool can switch between being light-harvesting antenna for photosystem I or photosystem II in plants and green algae. This switch, termed state transitions, involves the reversible phosphorylation of the mobile chlorophyll a/b-binding proteins, which is regulated by the redox state of the plastoquinone mediating electron transfer between photosystem I and photosystem II [19]. Therefore, the mechanisms underlying the differential expression of this protein in different barley genotypes should be further explored. Transketolase is an enzyme of both the pentose phosphate pathway in all organisms and the Calvin cycle of photosynthesis in plants [3], chloroplast transketolase was up regulated in XZ5 while non expression in XZ54 and ZAU3 under drought stress (spots A14 and B2, Tables 1, 2, and 3), indicating that carbon metabolism was also induced under drought stress. Os05g0405000, pyruvate orthophosphate dikinase (spot D2, PPDK) is a critical enzyme for C4 photosynthesis, providing the primary acceptor for fixation of bicarbonate in mesophyll cells. A key feature of C4 photosynthesis is the use of Phosphoenolpyruvic acid (PEP) as the initial acceptor of bicarbonate, and to allow this the formation of PEP is normally catalysed by PPDK [20]. PPDK is also present in C3 plants. Chastain et al [21] reported that C3 PPDK in leaves of several angiosperms and in isolated intact spinach chloroplasts undergoes light-/dark-induced changes in phosphorylation state in a manner similar to C4 dikinase, and likely represents the ancestral isoform of this unusual and key C4 pathway regulatory “converter” enzyme. The PPDK was up regulated in XZ5 while down regulated in XZ54 and ZAU3 after Five days of recovery, indicating PPDK may play an important role in maintaining higher photosynthesis in XZ5 in response to recovery.

### Energy related proteins

ATP synthase CF1 beta subunit (spot D5, Table 2) and ATP synthase beta subunit (B3, Table 1) were expressed at the highest level in drought-tolerant wild genotype XZ5 over the XZ54 and ZAU3, whereas it was down-regulated in XZ54 under drought stress at 10 % or 4 % soil moisture content (SMC). In plants ATP synthase is present in chloroplasts and integrated into thylakoid membrane; the CF1-part sticks into stroma, where dark reactions of photosynthesis and ATP synthesis take place. The ATP-dependent synthase/protease plays an essential role in controlling the availability of short-lived regulatory proteins and in removing abnormal or damaged proteins. It has been reported that these enzymes play a critical role in the removal of damaged proteins and in the fine control of some key cellular components, combining a peptidase and a chaperone activity [22]. Therefore drought induced higher expression of ATP synthase in XZ5 could be an attribute for its high drought tolerance. Enhanced abundance of ATP-synthesis related proteins under stress conditions, such as salinity and drought, has previously been shown by several studies [23, 24], although contrasting findings have also been reported [25]. In addition, an increase of the level of such proteins, such as ATP synthases, has been implicated to play an indirect role on ion homeostasis under salt stress, where elevated ATP levels drive H^+^-ATPases to generate a proton gradient which, in turn, drive Na^+^/H^+^ antiporters to translocate excessive Na^+^ and Cl^−^ ions into the vacuole [26]. Although the role of ion transporters in drought stress response is not fully understood, their involvement in drought stress is well-established. It was showed that induction of the protein in tolerant barley genotypes may alleviate water-deficit stress by increasing ATP supply to meet increased stress-related energy demand [27].

### Stress response related proteins

Twelve of the identified proteins are involved in stress response: heat-shock protein (spot C11), glutathione S-transferase 1 (spot A6), putative ascorbate peroxidase (spots C8 and C10), Thioredoxin M-type, chloroplast precursor (Trx-M) (spot C13), putative mono-dehydroascorbate reductase (spots B4 and D6), ATP-dependent peptidase/ATPase/metallopeptidase (spots A11, C9, D4) and melanoma-associated antigen p97 (spots A3 and C2). These proteins were up-regulated in response to drought stress in the drought tolerant wild genotype XZ5, but down-regulated/not expressed in the sensitive wild genotype XZ54 (Tables 1, 2 and 3). Heat shock protein (HSPs)/chaperons, the responsibility for protein folding, assembly, translocation and degradation, can not only stabilize proteins and membranes, but also assist refolding process of protein under stress conditions [28]. Acting as molecular chaperones, HSPs are responsible for protecting cells from stress injury, and its major function was characterized as stabilizing protein folding as well as preventing denaturation and aggregation which induced by stress [29]. According to the genomic investigations, Hsp has a higher expression level at both the mRNA and protein level in drought stress tissues [30]. The glutathione S-transferases (GST) represents a major group of detoxification enzymes [31], which catalyses the glutathione-dependent detoxification reactions and the reduction of hydroperoxides. In the present study, drought stress induced the increased GST (spot A6, Table 1) in XZ5, while no expression in sensitive genotype XZ54. GST were found to increase during slow drought or rehydration following rapid drought of the drought tolerant moss (*Tortula ruralls*) [32]. Furthermore, GST may act as binding proteins that sequestrate flavonoids (e.g. anthocyanins) in the vacuole for protection against environmental stresses [33]. The increased level of heat shock protein (spot C11, Table 3) and GST (spot A6, Table 1) in the tolerant genotype (XZ5) in the present study indicates their roles in drought stress tolerance in barley.

In addition, plants exposed to drought generate significantly reactive oxygen species (ROS), which, on one hand can cause damage to cellular components, and on the other hand, can act as signaling molecules for stress responses [34]. Plants can also regulate ROS level through antioxidant defense enzyme scavenging them such as ascorbate peroxidase (APX), mono-dehydroascorbate reductase (MDHAR) and superoxide dismutase (SOD). Two of the proteins identified in this study are involved in anti-oxidative system: putative APX (spots C8 and C10) and putative MDHAR (spots B4 and D6) (Tables 2 and 3). APX and MDHAR are two enzymes involved in the ascorbate-glutathione cycle, an efficient antioxidant system in the detoxification of ROS [35]. The cycle maintains a ratio of a reduced per oxidized ascorbic acid (AsA) and glutathione (GSH) for proper scavenging ROS in plant cells [36]. Secenji [37] found that the transcript level of relative *APX* gene was obviously changed in two genotypes of wheat when exposed to drought. Similarly, cytosolic *APX1* gene showed a higher expression in both genotypes. However, cytosolic *APX2* gene was up-regulated only in the drought-tolerant genotype. Thylakoid *APX* gene increased in the drought-tolerant genotype, while stromal *APX2* gene was found with higher expression levels in the drought-sensitive cultivar [37]. The increased or stable activities of APX and MDHAR observed in XZ5 under drought stress could maintain ROS detoxification, and under control condition the expression abundance of them were higher in drought tolerant genotype XZ5 than sensitive XZ54. Generally, the increased APX and MDHAR activities in the leaves of barley under drought stress may help maintain levels of AsA and GSH, the two important antioxidants against ROS toxicity [38]. Responses of the ascorbate-glutathione cycle to drought stress and recovery indicate that a co-regulated antioxidant mechanism could develop in barley.

FtsH (ATP-dependent peptidase/ATPase/ metallopeptidase) is a unique protease that is both membrane bound and ATP dependent and acts against integral membrane proteins. It plays pivotal roles in the quality control of membrane proteins by rapidly eliminating abnormal membrane proteins in prokaryotic organisms as well as in the mitochondria and the chloroplasts of eukaryotic cells. In vitro studies have suggested that thylakoid FtsH protease is involved in the degradation of unassembled proteins [39], and in the turnover of the D1 protein of the PSII reaction center in the context of its repair from photoinhibition [40]. It should be noted that a similar role for FtsH has been demonstrated in cyanobacteria. FtsH hexamers are further shown to be heteromeric, containing ‘Type A’ (FtsH1, FtsH5) and ‘Type B’ (FtsH2, FtsH8) subunits [41]. Mutant studies showed that the presence of at least one protein from each type is essential for FtsH hexamers to accumulate and function properly [42]. FtsH1 was not changed in XZ5 while down regulated in XZ54 and ZAU3 after nine day drought stress (SMC 10 %) and two and five days of recovery, illustrating that FtsH1 may have ability to repair damage to the D1 polypeptide during stress.

### Amino-acid biosynthesis and metabolism related proteins

Four of the identified protein spots are involved in amino-acid biosynthesis, including adenosylhomocysteinase (A2), elongation factor G (spots B5, C6 and C7) (Tables 1, 2, and 3), which were expressed at the highest level in XZ5 over the XZ54 and ZAU3, whereas it was down-regulated in XZ54 under drought stress (SMC 10 %) and two days of recovery. Elongation factors are a set of proteins that facilitate the translational elongation, the steps in protein synthesis from the formation of the first peptide bond to the formation of the last one [43]. The other 3 identified protein spots are involved in metabolism: glyceraldehyde-3-phosphate dehydrogenase 2 (GAPDH2, spot C4), Os02g0739600 putative pyruvate dehydrogenase E1 alpha subunit (spot C5), putative ankyrin repeat domain protein 2 (spot A13) (Tables 2 and 3). GAPDH has been considered to be primarily a housekeeping enzyme involved in the glycolytic pathway, catalyzing the NAD-dependent conversion of glyceraldehyde-3-phosphate into 1,3-diphosphoglycerate. Moreover, expression of GAPDH in plants leads to decreased constitutive ROS levels and enhanced tolerance to heat shock-induced cell death, which was up regulated in XZ5 and down regulated in XZ54 and totally inhibited in ZAU3 after two days of recovery. In plants, pyruvate dehydrogenase complexs (PDCs) are present in both mitochondria and chloroplasts. Both complexes consist of several copies each of at least four enzymes, which together convert pyruvate to acetyl-CoA under release of CO_2_ and production of NADH [44]. Whereas the mitochondrial complex mainly provides substrates for the citric acid cycle, the chloroplast homologue produces precursors for fatty acid biosynthesis [45]. The ankyrin repeat (ANK) protein family plays a crucial role in plant growth and development and in response to biotic and abiotic stresses [46]. Most of the *ANK* genes in tomato genome were up-regulated or down-regulated by abiotic stresses such as salt, heat, drought or wounding [47].

During the past decade, large-scale DNA sequencing efforts have produced a wealth of information about genomes. An important step in the analysis of gene information is deciphering the complete coding potential or protein coding sequence (CDS) region of each gene. Using pairs of specific primers, we cloned the length CDS region of the two genes, *rbcL* (Ribulose-1,5-bisphosphate carboxylase/oxygenase large subunit; Gene Bank Accession Numbers: gi|31087908|gb|AY137456.1) and *Trx-M* (Thioredoxin M-type, chloroplast precursor; Gene Bank Accession Numbers: AK360709.1). Comparing the nucleotide sequences, we found that nucleotide sequences performed differently in XZ5 and XZ54. The differences in *rbcL* and *Trx-M* gene sequences between XZ5 and XZ54 contained 5 and 2 SNPs, respectively. There were 2SNPs resulted in missense mutation bothin the *rbcL* and *Trx-M* genes. Although synonymous mutations cannot affect coding amino acid sequence, it induces the enhancement of exon splicing. Thus translation rate and the half-life of mRNA molecules will be affected, in this way influencing protein expression levels, and even altering the spatial structure of protein. If the structure of a particular protein changed, its normal function may be lost. We speculate that the SNPs up-regulated the protein expression of XZ5, and down-regulated in XZ54. In addition, in *Arabidopsis*, at least four Trx isoforms (*m*, *f*, *x*, and *y*) have been found in the chloroplast [48, 49]. Mohammed and Madhur [50] suggested that *OsTrx3* may participate in regulating the enzyme activities in photosynthesis (Calvin cycle) and metabolism (FBPase, MDH, and FTR) in *Oryza sativa*. Similar results have also been found in pea chloroplast and roots [51–54]. Plants frequently encounter external stresses conditions that adversely affect growth and development. Abiotic stresses trigger a wide range of plant responses, from alteration of gene expression and cellular metabolism to changes in plant growth rates and crop yields. A high expression indicated that the genes may play an important protective role against stressful hormone and light/dark conditions. In contrast, a low expression of the important genes in response to abiotic/hormone treatments, although possibly functioning as a survival strategy, may make the plants more vulnerable to those treatment conditions [50]. Our findings showed that Trx-M (Spot C13) was up-regulated in response to drought stress in the drought tolerant wild genotype XZ5, but down-regulated/not expressed in the sensitive wild genotype XZ54 (Table 2), indicating that *Trx-M* gene may play an important protective role against drought stress in the drought tolerant wild genotype XZ5. Further studies are warranted to elucidate the underlying molecular and metabolic pathways to better understand the mechanisms involved in drought-tolerance of wild barley and provide new molecular resources to develop more drought-tolerant crops.

## Conclusions

It is the first study to identify drought-responsive proteins in Tibetan wild barley (XZ5) using a proteomic approach. Because Tibetan wild barley XZ5 and XZ54 are the two contrasting genotypes with different drought tolerances, the drought-regulated proteins identified using comparative proteomics will provide a good foundation to elucidate the mechanisms involved in drought-tolerance in Tibetan wild barley. Here we found that barley plants respond to drought stress by changing the expression pattern of several proteins involved in photosynthesis, stress response, amino acid synthesis, energy and metabolism. The drought tolerance-associated proteins may be key factors that regulate these pathways. It is important to note that 20 of the 38 identified drought tolerance-associated proteins, up-regulated in XZ5 and simultaneously down-regulated/unaltered in XZ54, may be specific proteins with important roles in drought tolerance/resistance in XZ5. Among them, 5 protein spots (i.e. spots A4, B1 and C3 type I chlorophyll a/b-binding protein b; spot B2 chloroplast transketolase; spot C2 melanoma-associated antigen p97), were markedly induced by drought in XZ5 but not expressed in XZ54 and ZAU3 by drought stress. This difference suggests that these specific proteins may play a crucial role in tolerance to drought stress in XZ5. Our findings highlight the significance of drought-tolerance-associated-specific-proteins, and provide an insight into proteomic basis for drought-tolerance in Tibetan wild barley which offers novel molecular resource for drought-tolerance. In addition, it is noteworthy that functions of some of these differentially expressed proteins and their direct involvement in stress tolerance are poorly understood.

## Additional files

Additional file 1: Table S1. Oligonucleotides used as primers for quantitative RT-PCR. Additional file 2: Table S2. RbcL and Trx-M gene PCR amplification primer information and PCR reaction conditions. Additional file 3: Figure S1. Representative two-dimensioal gel electrophpresis maps of leaf proteins of XZ5 after different days of control condition. Additional file 4: Figure S2. Representative two-dimensioal gel electrophpresis maps comparing XZ54 leaf proteins isolated from normal (A-D) and drought for 9 day (soil moisture content, SMC 10 %, E), 20 day (SMC 4 %, F), and after 2 day re-watering (60–80 % water holding capacity, G) and 5 day re-watering (H), respectively. Additional file 5: Figure S3. Representative two-dimensioal gel electrophpresis maps comparing ZAU3 leaf proteins isolated from normal (A-D) and drought for 9 day (soil moisture content, SMC 10 %, E), 20 day (SMC 4 %, F), and after 2 day re-watering (60–80 % water holding capacity, G) and 5 day re-watering (H), respectively. Additional file 6: Figure S4 ‘Spot view’ of the abundance of differentially expressed proteins (indicated with green circles) in leaves of three barley genotypes XZ5, XZ54 and ZAU3 at 2 days (A) and 5 days (B) of recovery following drought stress. Additional file 7: Figure S5. The partial-length CDS of *rbcL* gene from three barley genotypes XZ5, XZ54 and ZAU3. Additional file 8: Figure S6. The full-length CDS of *Trx-M* gene from three barley genotypes XZ5, XZ54 and ZAU3. Additional file 9: Figure S7. Shoot biomass of XZ5, XZ54 and *cv* ZAU3 after 5 day recovery (a), and plant morphology at SMC 4 % (b). The bar represents the standard error.

## Abbreviations

ACN: Acetonitrile
ANK: Ankyrin repeat
APX: Ascorbate peroxidase
FtsH: ATP-dependent peptidase/ATPase/ metallopeptidase
GAPDH: Glyceraldehyde-3-phosphate dehydrogenase
GST: Glutathione S-transferases
MDHAR: Mono-dehydroascorbate reductase
PEP: Phosphoenolpyruvic acid
PDC: Pyruvate dehydrogenase complex
PPDK: Pyruvate orthophosphate dikinase
*rbcL*: Ribulose-1,5-bisphosphate carboxylase/oxygenase large subunit
RuBisCO: Ribulosebisphosphate carboxylase
ROS: Reactive oxygen species
SMC: Soil moisture content
SOD: Superoxide dismutase
*Trx-M*: Thioredoxin M, chloroplast precursor
