# Supplementary material for: Comparative proteomic analysis of drought tolerance in the two contrasting Tibetan wild genotypes and cultivated genotype
Source: BMC Genomics. 2015 Jun 5;16(1):432. doi: 10.1186/s12864-015-1657-3 (PMC4456048; doi:10.1186/s12864-015-1657-3)
Supplement: Additional file 1: Table S1. — Oligonucleotides used as primers for quantitative RT-PCR. [file 12864_2015_1657_MOESM1_ESM.doc]

**Table S1.** Oligonucleotides used as primers for qRT-PCR.

| Protein | Primer orientation | Primer sequence(5’-3’) | Amplified size |
| --- | --- | --- | --- |
| Ribulosebisphosphate carboxylase large chain precursor | Forward | AGCCCTGGCTTCGTCTTGA | 121 |
|  | Reverse | CACTGCATCGGCACAGAGTT |  |
| ATP synthase beta subunit | Forward | TTCCGTGATGCCGAAGGA | 119 |
|  | Reverse | TGGTATCCCACAGCAGATGGA |  |
| Heat-shock protein | Forward | GCTGTACGAGACTGCCCTGAT | 119 |
|  | Reverse | TGCCGGATCTACCCCATCT |  |
| Os05g0405000 | Forward | GCAGCTCAAGGTCATGGCTAA | 119 |
|  | Reverse | TCTCGTCAGAAGCAAAGAACATGT |  |
| ATP synthase CF1 beta subunit | Forward | GAGAATGTGGTCCGCACCAT | 120 |
|  | Reverse | AATACGCCCAAGAGTCGCTCTA |  |
