# Supplementary material for: Comparative proteomic analysis of drought tolerance in the two contrasting Tibetan wild genotypes and cultivated genotype
Source: BMC Genomics. 2015 Jun 5;16(1):432. doi: 10.1186/s12864-015-1657-3 (PMC4456048; doi:10.1186/s12864-015-1657-3)
Supplement: Additional file 2: Table S2. — RbcL and Trx-M gene PCR amplification primer information and PCR reaction conditions. [file 12864_2015_1657_MOESM2_ESM.doc]

**Table S2**  RbcL and Trx-M gene PCR amplification primer information and PCR reaction conditions

| Genotype | Gene | Primer name | Primer sequence(5’-3’) | PCR **reaction conditions** |
| --- | --- | --- | --- | --- |
| XZ5  XZ54 | *rbc*L | rbcL 1 (Forwoard) | TAGACCCTGTTATTGTGAGA | 94℃/ 1 min-30 cycles (98℃/ 10 sec-51.7℃/ 30 sec-72℃/ 2 min)-72℃/ 10 min |
|  |  |  |  | 94℃/ 1 min-30 cycles (98℃/ 10 sec-57.1℃/ 30 sec-72℃/ 2 min)-72℃/ 10 min |
| ZAU3 |  | rbcL2 (Reverse) | GAATTTGATCGCCTTCC | 94℃/ 1 min-30 cycles (98℃/ 10 sec-57.1℃/ 30 sec-72℃/ 2 min)-72℃/ 10 min |
| XZ5 | *Trx-M* | Trx-M 1 (Forwoard) | GCAATGGCCTTGGAGA | 94℃/ 1 min-30 cycles (98℃/ 10 sec-48℃/ 30 sec-72℃/ 2 min)-72℃/ 10 min |
| XZ54 |  |  |  | 94℃/ 1 min-30 cycles (98℃/ 10 sec-50℃/ 30 sec-72℃/ 2 min)-72℃/ 10 min |
| ZAU3 |  | Trx-M 2 (Reverse) | GCTGCCGATGTACTTGTC | 94℃/ 1 min-30 cycles (98℃/ 10 sec-52℃/ 30 sec-72℃/ 2 min)-72℃/ 10 min |
