# Supplementary material for: Comparative proteomic analysis of drought tolerance in the two contrasting Tibetan wild genotypes and cultivated genotype
Source: BMC Genomics. 2015 Jun 5;16(1):432. doi: 10.1186/s12864-015-1657-3 (PMC4456048; doi:10.1186/s12864-015-1657-3)
Supplement: Additional file 3: Figure S1. — Representative two-dimensioal gel electrophpresis maps of leaf proteins of XZ5 after different days of control condition. [file 12864_2015_1657_MOESM3_ESM.doc]

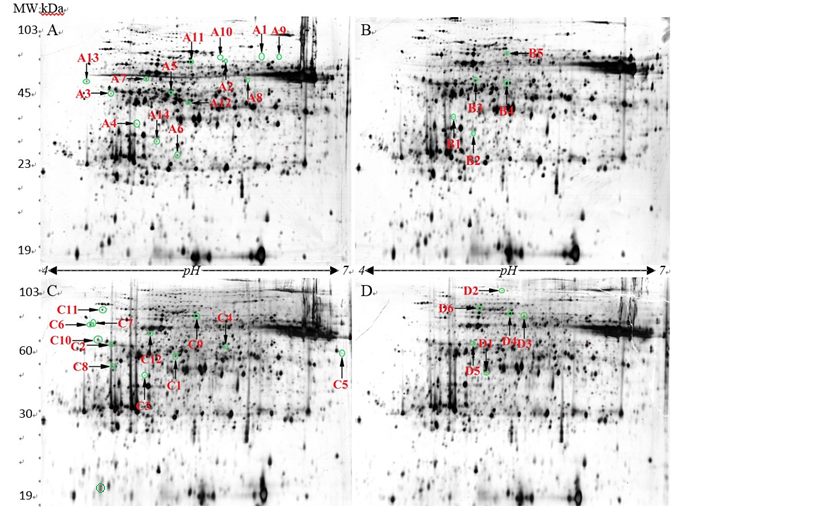


**C13**

**Figure S1. Representative two-dimensioal gel electrophpresis maps of leaf proteins of XZ5 after different days of control condition.** The proteins were isolated from the leaves of XZ5 plants under 60-80% water holding capacity, after 9 day (A), 20 day (B) drought experience for the drought treatment, and after 2 day re-watering (C) and 5 day re-watering (D), respectively. Total proteins were extracted and separated by 2-DE. In IEF, 100 mg proteins were loaded onto pH 4–7 IPG strips (24 cm, linear). SDS-PAGE was performed with 12.5% gels. The spots were visualized by silver staining. Differentially accumulated protein spots are indicated by green sashes, and marked with arrows and numbers.

C13
