# Supplementary material for: Comparative proteomic analysis of drought tolerance in the two contrasting Tibetan wild genotypes and cultivated genotype
Source: BMC Genomics. 2015 Jun 5;16(1):432. doi: 10.1186/s12864-015-1657-3 (PMC4456048; doi:10.1186/s12864-015-1657-3)
Supplement: Additional file 5: Figure S3. — Representative two-dimensioal gel electrophpresis maps comparing ZAU3 leaf proteins isolated from normal (A-D) and drought for 9 day (soil moisture content, SMC 10 %, E), 20 day (SMC 4 %, F), and after 2 day re-watering (60–80 % water holding capacity, G) and 5 day re-watering (H), respectively. [file 12864_2015_1657_MOESM5_ESM.doc]

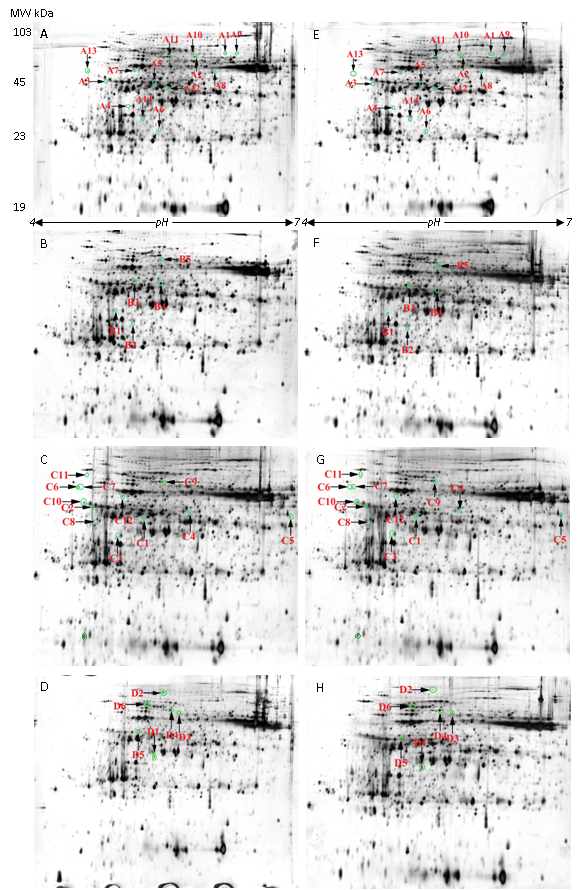


**C13**

**C13**

**Figure S3** Representative two-dimensioal gel electrophpresis maps comparing ZAU3 leaf proteins isolated from normal (A-D) and drought for 9 day (soil moisture content, SMC 10%, E), 20 day (SMC 4%, F), and after 2 day re-watering (60-80% water holding capacity, G) and 5 day re-watering (H), respectively. Total leaf proteins were extracted and separated by 2-DE. In IEF, 100 g of proteins were loaded onto pH 4–7 IPG strips (24 cm, linear). SDS-PAGE was performed with 12.5% gels. The spots were visualized by silver staining. Differentially accumulated protein spots are indicated by green sashes.
