# Supplementary material for: Comparative proteomic analysis of drought tolerance in the two contrasting Tibetan wild genotypes and cultivated genotype
Source: BMC Genomics. 2015 Jun 5;16(1):432. doi: 10.1186/s12864-015-1657-3 (PMC4456048; doi:10.1186/s12864-015-1657-3)
Supplement: Additional file 6: Figure S4 — ‘Spot view’ of the abundance of differentially expressed proteins (indicated with green circles) in leaves of three barley genotypes XZ5, XZ54 and ZAU3 at 2 days (A) and 5 days (B) of recovery following drought stress. [file 12864_2015_1657_MOESM6_ESM.doc]

**(A)**


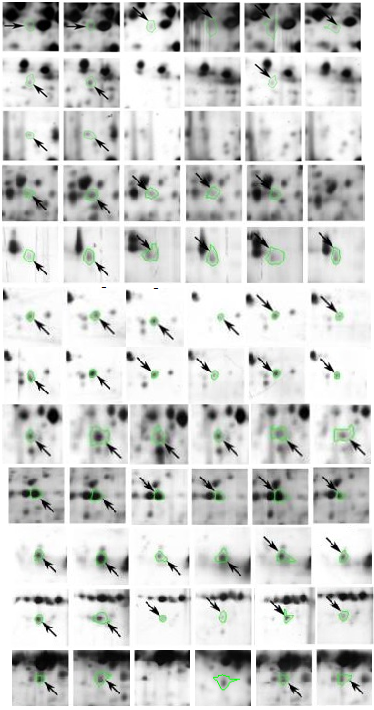


**Control Drought Control Drought Control Drought**

**XZ5 XZ54 ZAU3**

Spot No.

C1

C2

C3

C4

C5

C6

C7

C8

C9

C10

C11

C12


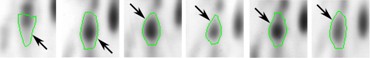


C13

**(B)**

**Control Drought Control Drought Control Drought**

**XZ5 XZ54 ZAU3**

Spot No.

**Figure S4** ‘Spot view’ of the abundance of differentially expressed proteins (indicated with green circles) in leaves of three barley genotypes XZ5, XZ54 and ZAU3 at 2 days (A) and 5 days (B) of recovery following drought stress. Protein spot ID refers to numbers in Fig. 1C-D and Tables 2 and 3.
