# Supplementary figures and images for: Comparative proteomic analysis of drought tolerance in the two contrasting Tibetan wild genotypes and cultivated genotype
Source: BMC Genomics. 2015 Jun 5;16(1):432. doi: 10.1186/s12864-015-1657-3 (PMC4456048; doi:10.1186/s12864-015-1657-3)

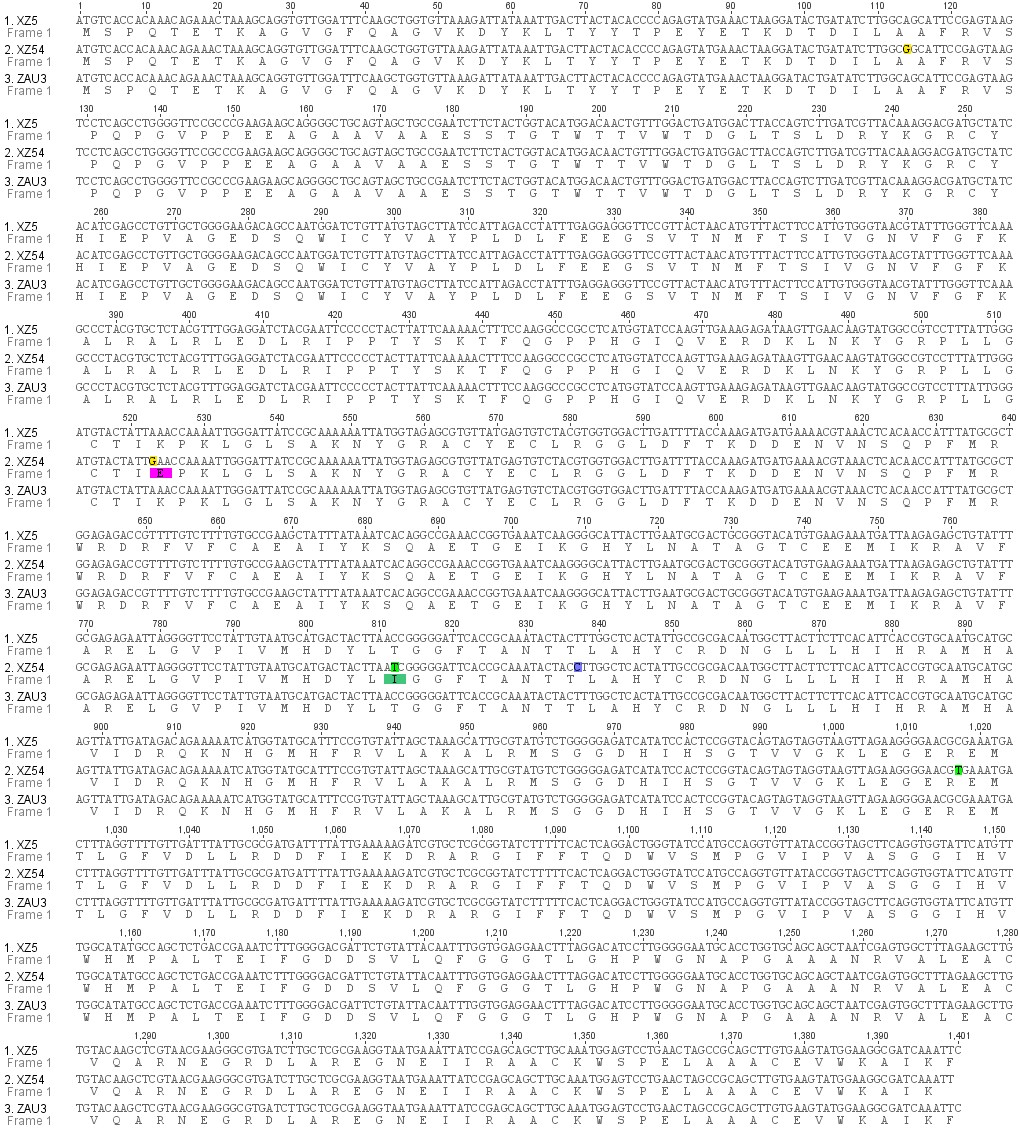


**Figure S5** The partial-length CDS of *rbcL* gene from three barley genotypes XZ5, XZ54 and ZAU3

Supplement: Additional file 7: Figure S5. — The partial-length CDS of rbcL gene from three barley genotypes XZ5, XZ54 and ZAU3. [file 12864_2015_1657_MOESM7_ESM.doc]

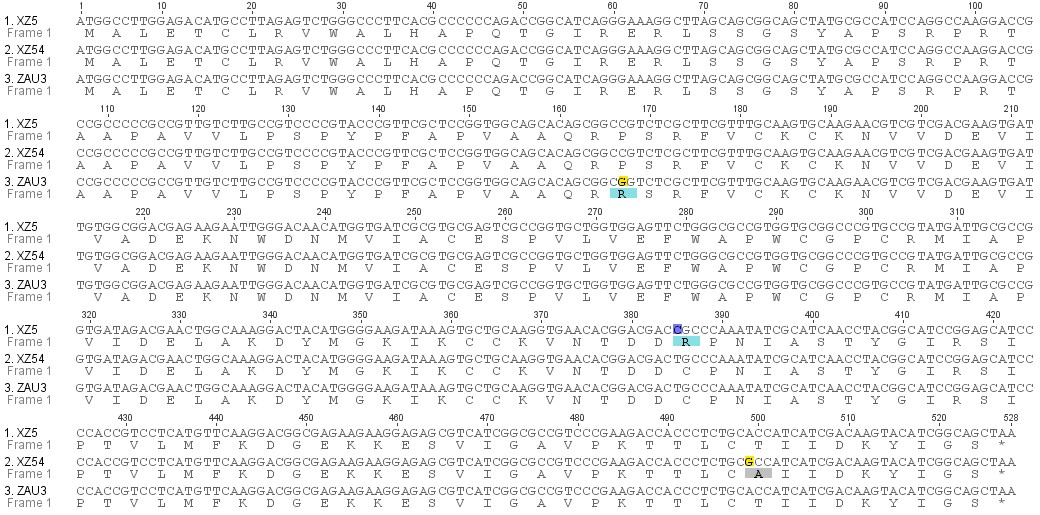


**Figure S6** The full-length CDS of *Trx-M* gene from three barley genotypes XZ5, XZ54 and ZAU3

Supplement: Additional file 8: Figure S6. — The full-length CDS of Trx-M gene from three barley genotypes XZ5, XZ54 and ZAU3. [file 12864_2015_1657_MOESM8_ESM.doc]
