# Supplementary material for: Comparative proteomic analysis of drought tolerance in the two contrasting Tibetan wild genotypes and cultivated genotype
Source: BMC Genomics. 2015 Jun 5;16(1):432. doi: 10.1186/s12864-015-1657-3 (PMC4456048; doi:10.1186/s12864-015-1657-3)
Supplement: Additional file 9: Figure S7. — Shoot biomass of XZ5, XZ54 and cv ZAU3 after 5 day recovery (a), and plant morphology at SMC 4 % (b). The bar represents the standard error. [file 12864_2015_1657_MOESM9_ESM.doc]

**(a)**


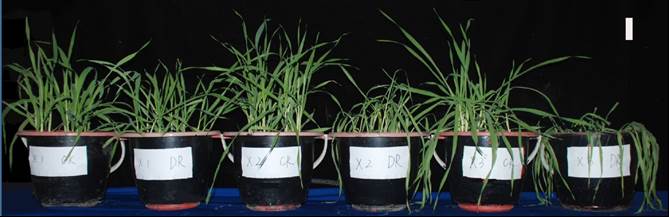


XZ5-Control XZ5-Drought XZ54-Control XZ54-Drought ZAU3-Control ZAU3-Drought

**(b)**

10 cm

**Additional file 9: Figure S7** Shoot biomass of XZ5, XZ54 and *cv* ZAU3 after 5 day recovery (a), and plant morphology at SMC 4% (b). The bar (a) represents the standard error.
